# Approaches of School Administrators toward Teachers with Different Types of Human Nature: The Cyprus Case

**DOI:** 10.3390/bs8080066

**Published:** 2018-07-25

**Authors:** Nedime Karasel Ayda, Nazım Kaşot, Ahmet Güneyli

**Affiliations:** 1 Department of Educational Management, Near East University, Nicosia 99138, North Cyprus; karaselnedime@hotmail.com; 2 Department of Science Teaching, Near East University, Nicosia 99138, North Cyprus; nazim.kasot@neu.edu.tr; 3 Nature Biomonitoring and Protection Research Center, Near East University, Nicosia 99138, North Cyprus; 4 Department of Turkish Education, Near East University, Nicosia 99138, North Cyprus

**Keywords:** human nature, importance of human nature in administration, management of different human natures, attitudes of school administrators

## Abstract

This study seeks answers to the questions regarding the types of human nature that primary school teachers possess from the perspective of primary school administrators and what their approaches are toward teachers with different types of human nature. The study is prepared using a case study model with the qualitative method. “Scenario analysis” is used to obtain data, and eight different scenarios prepared within this context were presented to school administrators. In total, 25 administrators during the 2017–2018 scholar year were selected based on purposive sampling and were contacted accordingly. In terms of the conclusions of the study, it can be seen that school administrators defined self-actualizing teachers with social human nature using positive adjectives, while they had different opinions in regard to defining teachers with a rational-economic human nature, and they defined teachers with a complex human nature with negative adjectives. In terms of the findings related to the attitudes of administrators toward different human natures, it was found that there are some administrators who display positive administrative behaviors (appreciating, rewarding, respecting, motivating etc.) as well as administrators who display negative administrative behaviors (punishing, being indifferent to incidents, imposing by using legal powers, etc.).

## 1. Introduction

Administration involves using resources effectively and productively based on previously established policies and decisions in order to reach the established goals of any organization [1]. School administration is the application of educational management in a limited space—the education environment [2]. School is the space where the decisions and policies taken within the educational organization are applied, and school administration is responsible for ensuring that educational services are functioning in the most effective way based on the goals of education [3]. The people who are tasked with these duties and responsibilities at schools, as well putting the school operation in order, are the administrators. Traditionally, a school administrator is considered as an administrator who procures resources related to the school within the scope of laws and regulations and ensures their organization and use. In conclusion, they work to realize the of school objectives and largely focus on the aims of preserving and sustaining the status quo. However, discussions on contemporary administrative theories emphasize the leadership behaviors of school administrators beyond administration. School administrators are expected to undertake contemporary leadership roles, such as designing the vision of the school, defining social relationships, ensuring the growth of the school, giving importance to school safety, and providing effective and coherent communication with the school employees [4,5].

Fundamentally, the effective power sustaining an administrator is seen as “authority.” Authority within an organization is the right to decide on the plans and policies for achieving organizational goals, mobilizing the organization and giving orders, inspecting the applications, executing actions, and defining how individuals will behave within his/her space of authority [6]. The level of contemporary educational management has evolved into a process of cooperation and understanding between the administrator and employees in order to reach the organizational goals, rather than a superior–subordinate relationship. Studies on school administrators show that teachers need leaders who actively participate in school activities, rather than administrators [7]. Leadership behaviors that administrators will display, and the will to demonstrate fidelity and responsibility toward the organization that teachers will display, are notions that are interrelated. Therefore, it is very important that administrators know the different personal characteristics and human natures of teachers working in their schools.

The basis of views on the theory of human nature can be traced to the 3rd and 4th Centuries B.C. According to the Sophist philosophers of that era, the natural impulse of those with powerful human nature was to rule those who were considered to be weak [8]. In the initial theories, which started with this view of the Sophists and attempted to explain human nature, anthropologists defined man as a being that structures meaningful symbols to explain his world and has the power to think [9], while those who believed in the metaphysical understanding defined human nature as qualities that are present from birth [10]. In another definition, Wrightsman described human nature as the general attitudes of an individual toward others [11].

Considering that administration is the action of realizing a specific goal or the action of completing work using human and material resources [12], it becomes clear that man is at the core of administration, and it is important to know the nature of people in order to manage them. Having knowledge about the nature of people that an administrator is required to manage is of vital importance for them to resolve possible conflicts and establish peace within the organization [13], to increase productivity in the educational institution and facilitate the achievement of organizational goals [14], to know how to motivate employees in the organization, and to enhance performance [15]. The limited number of studies on human nature that can be found in the Turkish literature [10,13,16,17] have concluded that school managers who understand and value teachers with different human natures are democratic, whereas managers who do not value them are autocratic managers. It has been displayed that school managers who value human nature are more effective in terms of organizational efficiency. In addition, it has been stated that determination of the perception of school managers concerning teachers with different human nature is very critical in terms of solving the problems in school management. It has been found that the perception of school managers concerning teachers with different human nature affects both their school management styles and their attitudes in human relations. In addition, it has been concluded that the terms in office of school managers at schools is effective in recognizing the teachers with different human natures [10,13,16,17].

In brief, this study aims to establish the circumstances surrounding recognizing human nature in administration, how the administrators are able to give meaning to the behaviors of people, and how they realize administrative processes suitable to the nature of the people they encounter. In this study, the objective is to establish the approaches of school administrators in primary schools in North Cyprus toward recognizing the human nature of teachers in their schools and their attitudes toward different human natures. Initially, primary school principals were asked to interpret the scenarios (sample situations) given to them in order to establish the natures of teachers in their schools and the number of teachers who were suitable for those scenarios; subsequently, they were asked to state their reactions and approaches when they experience such scenarios. The findings obtained from the research are thought to provide a contribution in relation to “the importance of administrators knowing human nature, in administration” and “establishing administrative approaches suitable to human nature.” Based on the findings of this research in North Cyprus, it can be said that awareness will be created in terms of informing school administrators on human nature, establishing suitable school administration strategies, and organizing in-service training.

## 2. Theoretical Framework

### 2.1. Development of the Notion of Human Nature and Basic Views

When analyzing the development of human nature, it can be said that in early periods, individuals could not isolate themselves from their environments, could not think about other individuals, and a belief was generally accepted by individuals within the tribe. Later, as a result of humans transitioning into more settled lives and the development of individual–environment relationships, individuals learned to isolate themselves from the communities to which they belonged [10]. According to Hobbes, during the development of human nature, passions were dominant before social life was developed, while ethical and political dimensions became predominant after moving into the social stage [18].

“Human nature”, which is an important notion from the social and psychological aspect, is explained with four basic points of views in studies conducted by social psychologists [19]. The first of these views is the “Hedonistic View”, which has been the most recognized aspect of human nature for the longest period of time. This view advocates that obtaining pleasure and contentment is decisive in human behavior [20]. The second view is the “rationalist view”. In this view, known as rationalism, it is proposed that selfish and antisocial human behaviors are managed by individual motivations. Hobbes states that in this view, the determinants of behavior are motivation and thought [21]. The “Machiavellist view” is the third view on the topic. This view advocates that human goals are connected to the motivation for life, and there are parallels to nature. According to this view, the determinants of human behavior are shaped based on the benefit for the individual. The last of the basic views on human nature is the “Existentialist view”. According to this view, people are unique in making life choices [22]. Furthermore, human behaviors are free and are formed based on the understanding of self-development.

### 2.2. Administration Theories and Human Nature

The first important contributions to administration were observed in the Sumerian civilization, who maintained written records and the development of steps such as “planning, organizing, and controlling”, which are considered as important milestones; subsequently, the Egyptians introduced the notions of decentralization, fair administration, and written petitions. Organization and administration continued with the development of administration as a science after the Industrial Revolution (20th Century) [23].

There are three basic theories in the history of administrative science, namely “Classical Administration Theory”, “Neo-classic Administration Theory”, and “Contemporary Theory”. These theories have evolved based on the differences in human nature, points of view toward human nature, and as reflections of changing opinions.

The advocates of Classical Administration Theory viewed people from the perspective of productivity and saw them as “economic and rational” beings [9]. In this theory, rules came into prominence, and it moved away from objectivity [12]. Neo-classical Administration Theory was developed as a reaction to Classical Administration Theory, and it was developed within the framework of notions such as human relationships, cooperation, social systems, and harmony within the organization [2]. This theory concentrated on the social and psychological structure of the human elements instead of economic factors [12]. The recently developed Contemporary Theory has emerged as a synthesis of the two previous ones. This theory not only concentrates on the working individual but on the individual–organization administration component as well. The representatives of this theory advocate that the interests of the individual and the organization are unified, and work must be completed in cooperation [12].

When analyzing these definitions, it can be said that human nature, which is the primary factor in the development of an organization and in the forming of organizational culture, is the basic determinant of the theories. All the theories developed on organization and administration were initially based on hypotheses related to human nature. While a “pessimistic” view is dominant in the Classical Administration Theory, the Neo-Classical Theory is dominated by an “optimistic” view, and there is a balanced situation in the Contemporary Theory. When theories on organization and administration are examined, it is observed that notions such as the human factor and human motivation, affectivity, rationalism, and administration come into prominence [9].

In the accumulation of studies on human nature, researchers have studied this topic under four basic models [11,24]. Human nature is accordingly divided into rational-economic human, social human, self-realizing human, and complex human.

(i) Rational-Economic Human: The basic statement defining this came from Taylor, who said “workers are motivated wholly by economic incentives”. Therefore, the way to motivate people with this nature is by using pay as both a reward and a punishment [12]. According to the rational-economic human theory, the duty of the leader is to use payment as a weapon to bring the productivity of employees to the optimum level, since they are primarily motivated by money [24]. This human nature is evocative of McGregor’s X theory, or the view that he calls the classical point of view of employees by companies (according to McGregor’s X theory, people are passive, lazy and stupid in general; they are unable to maintain discipline over their actions [11]). Aydın explains this in the following way: people cannot keep themselves under control, therefore authority is needed to keep them under control, to motivate them, and to direct them toward the goals of the organization [12]. This power, according to rational-economic human nature, is their payment.

(ii) Social Human: According to Mayo, the need to be accepted and liked by peers in an organizational environment is more important than the economic incentives provided by the administration. Therefore, it is advocated that individuals with this human nature are fundamentally motivated by their social needs. With the range of work developing as a result of the Industrial Revolution, it became very difficult for people to complete all their tasks and the need for division of labor became inevitable. As a result of working together, social networking skills within the work environment have developed and socializing has increased in prominence [16]. In explaining this human nature, Schein stated that working people try to realize the wishes of the administration in order to gain social acceptance. Mayo also advocated that people with this nature are more sensitive to social pressure rather than material incentives issued by the administration.

(iii) Self-realizing Human: This is a model advocating that human needs have a hierarchical structure and that moving up to higher level needs as the lower ones are met is part of human nature [12]. According to this model, people desire to become perfect, autonomous, and independent in their work. Individuals with this human nature have the skills required for self-motivation and self-control.

(iv) Complex Human: The inclination of people to change from time to time or from situation to situation has resulted in complex human nature having a place among the other models. People of this nature are not just complex within themselves, but they are also changeable. As Aydın cited, people have many incentives to have a certain hierarchical structure within themselves based on their importance [12]. These incentives can show variability in different organizations, or at different sub-levels of the same organization. When people’s changing incentives come together with preemptive needs and organizational experiences, new incentive elements may form. Administrators who believe in the complexity of people have to be sensitive toward the personal differences, fears, and skills of their employees. Instead of seeing individuals’ differences as unwanted realities, administrators should endeavor to determine the reasons behind those differences and give priority to how they can be managed [10].

These varieties of nature are considered as human nature and are classified under the four models shown above; however, McGregor classified them as two opposite assumptions and named them the “X and Y Theory”. According to this classification, the human nature in which individuals who do not like working and avoid responsibility, where the view that punishment and outside control is needed (in other words, the pessimistic view where punishment and fear prevails), is called “X Theory”. On the other hand, “Y Theory” describes the human nature in which the view of self-controlling, passionate, and creative individuals who accept their responsibilities prevails [25]. Many researchers have listed and classified the characteristics of the types of people described in these theories [9,11,12,26,27,28,29]. The “Y Theory” proposed by McGregor in relation to human nature has been supported throughout historical development by Lock, Hume, Voltaire, Rousseau [12]. Additionally, Fromm and Rainer conducted studies supporting this theory [26,30].

Studying the four models of human nature and the X-Y Theory, Elfil found that administrators embracing the X Theory are authoritative and interfering, while those embracing the Y Theory display democratic and participatory behaviors [31].

This study will assess the characteristics of school administrators based on their attitudes toward teachers with different human natures, and thus, it will be established to which educational administrative theory they are more inclined.

## 3. Materials and Methods

### 3.1. Problem Statement and Sub-Problems

The problem sentence of this study was formulated as “What are the approaches of school administrators toward teachers with different human natures working at primary schools in North Cyprus?” The two sub-questions shown below were also designed in relation to this problem statement:

1. What human nature characteristics do primary school teachers have in the eyes of school administrators?
2. What are the attitudes of school administrators toward the different human natures of primary school teachers?

### 3.2. Model of the Study

This study uses a qualitative research approach. The qualitative research approach has important features such as sensitivity to the environment, the participatory role of the researchers, a holistic approach, flexibility in the research design, perceptions can be put forward, and inductive analysis is ensured [32].

The “case study” method of qualitative research was used as the basis, and an “entwined single case” design, which allows more than one analysis within the single case design (according to Yin, 2009), was used [33]. The case examined in this research comprises primary school teachers under the Ministry of National Education of North Cyprus, while the analysis units are the different human natures and the attitudes of school administrators toward these different human natures.

### 3.3. Case of the Study: North Cyprus Education System

The education system in Northern Cyprus is affected by the Turkish education system. Education management in Northern Cyprus has a centralized structure, and the management of schools is performed by school principals in particular and the Ministry of National Education in general. Pre-school education is not obligatory in Northern Cyprus. However, the period until the end of secondary school (from 1st grade to 14th grade) and the period covering the ages between seven and 14 is obligatory. The education is provided in the Turkish language at school, but foreign languages are also taught starting at the elementary school level. The teachers who provide education at schools are chosen from those who have studied at education faculties for at least four years after high school. Teachers are chosen and assigned based on a central written and verbal evaluation process organized by the state. Education is not the only duty of teachers in Northern Cyprus. The fundamental duties of teachers in addition to education are ensuring that national values are entrenched and developed in students, raising them as good citizens and people, and as individuals who will provide benefits to the social, as well as the cultural and economic development of the country. School principals have to ensure that teachers at the school perform their duties and that all affairs of the school are properly conducted. School principals are chosen and assigned by the Ministry of National Education from eligible candidates with a minimum of 10 years of teaching experience and successful results obtained from the written examination organized by the state. The primary duties of school principals can be summarized as follows: conducting the management affairs of the school, ensuring that official documents, statistics, records and books are kept and preserved, organizing school activities and ensuring that school programs are implemented, establishing the necessary relationships between the school and students’ parents, assisting the functioning of the parent-teacher association, and protecting the various buildings, facilities, and fixed assets of the school. If school principals are inadequate or unable to offer solutions to problems, the Ministry of National Education assigns education supervisors (inspectors) in order to review the activities and problems experienced in the school. These supervisors have the authority to supervise the school principal and the school administration under his/her leadership [34].

### 3.4. Sampling of the Research

Administrators working at state primary schools under the Ministry of National Education of North Cyprus formed the sample of the research. Twenty-five school administrators were reached, and the sample was formed accordingly. Five of those school administrators have Master’s degree in educational management, and two of them have a master’s degree in various other fields of educational sciences. Therefore, the “purposive sampling” approach is suitable in this instance. Purposive sampling is defined as the in-depth examination of situations in which it is expected that rich data will be obtained [32]. The administrators selected for the purposive sampling were those serving in different parts of the island with whom the researchers were already acquainted, and they were therefore easily accessible, making them suitable for “easily accessible case sampling”.

### 3.5. Data Collecting Tool

Scenarios were used to determine the natures of the teachers and the attitudes of administrators toward different natures. Scenarios are not used to predict the future, but as notions reflecting different opinions for the past and for future developments and serve as alternative assumptions for the future. Scenarios are useful ways for providing a holistic point of view, perceiving things from a different perspective, and for answering questions [35]. The scenario technique has an important place in the contemporary administration literature [36].

The scenarios and scenario analyses used in the research are taken as the basis for the future analysis process by taking into account possible alternative conclusions [37]. Scenarios are important for constructing a story about how the future will be shaped [38].

The scenarios prepared by the researchers were sent to three experts, two of whom are experts in the field of educational management, and one in Turkish Language. Later, a pilot application of the scenarios was conducted with four primary school administrators. Thus, the scenarios were given their final shape. The scenarios used in the study and the human natures they represent were structured as below:

Scenario-1: (Rational-Economic Human Nature)

“Y1 Teacher is 50 years old and has been a classroom teacher for 25 years. This teacher is working in your school is legally in “A Teacher” category and has a maximum workload of 20 h a week. For some reason (school team attending a competition, sickness, apology leave, overseas leave, assignment by the administrator etc.), there is a special course teacher shortage at the school. This shortage affects the class of your Y1 teacher as well. Although he does not initially accept to fill in for the shortage, after learning that overtime will be paid, Y1 teacher accepts to work during his free period.”

Scenario-2: (Social Human Nature)

“Y2 teacher has been working in your school for a very long time. He takes responsibilities during days that are important for the teachers (New Year, Teachers’ Day, commencement of summer holiday, just before semester break, graduation period etc.) and organizes entertainment on those days.”

Scenario-3: (Complex Human Nature)

“Y3 teacher has been working in your school for 2 or more years. Although he/she regularly signs the “teacher meetings decision records”, documenting in writing that he/she accepts the decisions taken, this year he/she orally informed the administration that he/she will not sign the records, without giving any reason.”

Scenario-4: (Self-Realizing Human Nature)

“Y4 teacher is always liked and appreciated by colleagues, parents, and administrators. He/she attends school almost every day throughout the academic calendar, and always stays at school during class hours unless a very serious problem arises. He/she is noticed for his/her work discipline and appraised by the inspectors.”

Scenario-5: (Self-Realizing Human Nature)

“Y5 teacher carries out his/her duty with great loyalty. Although there is no obligation, he/she coaches his students who are behind in their schoolwork during his/her free time in the afternoon, and gives them supplementary lesson notes from his own books. In addition, he/she attends educational courses and conferences with an understanding of life-time learning, without an aim of professional promotion, and improves him/herself.”

Scenario-6: (Complex Human Nature)

“Y6 teacher has been working in your school for a long time. Every year, when classes are distributed among teachers at the beginning of the school year, he/she disputes with the school administration about teaching elder (4th or 5th grade) students, and he/she insistently gets one such class. This year, when you were distributing classes, although you gave him/her a high grade class, he/she stated after the staff meeting that he/she did not want that class and that this year he/she wanted to teach a lower grade class.”

Scenario-7: (Rational-Economic Human Nature)

“Y7 teacher did not want to put up any lesson sheets on the clipboard in the classroom on the grounds that “School year preparation allowance was not paid.” After the “preparation allowance” was paid in October, it was observed that the clipboards in his/her classroom were filled.

Scenario-8: (Social Human Nature)

“Y8 teacher works at your school and is helping to manage the web page created for your school by the Ministry of National Education. At the beginning, when no other teacher had knowledge about this application, Y8 teacher stated that he/she could take the responsibility without hampering his duties at school. He/she contacted the authorities at the ministry and acquired knowledge about running a web page and has taken this responsibility upon themselves every year voluntarily.”

### 3.6. Data Collection

The research was conducted during the 2017–2018 Academic year, between 2 October 2017 and 4 December 2017, with 25 primary school administrators from the Ministry of National Education of North Cyprus. The administrators were given forms consisting of two sections and were asked to complete them accordingly. First, the administrators were asked to complete the first section of the forms, which pertained to their personal information. Then, they were asked to read the above scenarios about “human nature” and state the number of teachers at their schools who corresponded to those scenarios. Finally, they were required to write in the blank places their attitudes toward those scenarios when they encountered them. Administrators completed the scenarios by thinking of and observing the behaviors of their teachers.

### 3.7. Data Analysis

Data analysis was completed using the “content analysis” method. Content analysis involves systematically analyzing written or oral material and quantifying what is said or written by coding the material [39]. The four stages developed by Miles and Huberman were used in content analysis, as shown below [40].

(a) Coding Data: Information obtained during the interviews was decoded and converted in a list by giving numbers to lines. Subsequently, the sections were coded to form meaningful wholes. The coding list was read by the researchers separately, and a “consensus” was reached. Topics with “differences of opinion” were discussed, and new arrangements were made. Differences of opinion that could arise among the researchers on the data obtained through interviews and their analysis were minimized; for that purpose, joint decisions were taken at every stage of the research.
(b) Finding the Themes: In the first stage, the established codes were collected under the structured categories. Thus, the researchers attempted to find common ground among the codes. Two themes were discussed for each scenario of the research, which are “defining the teacher”, and “attitude of the administrator toward the teacher”.
(c) Arranging Data Based on Codes and Themes: Each administrator was given a number by the researchers in order to convey the opinions of the participants to the readers, starting with number 1. In terms of the participant opinions for the interview questions in the findings, administrators were mentioned with these codes (i.e., Y-1), and citations were made from administrator opinions related to each finding with the code of the cited administrator given in parenthesis.
(d) Defining and Interpreting Findings: Findings obtained as a result of interviews with the participating administrators and interpretations of these findings are presented in this section. Findings are given in detail with citations, thus strengthening the validity of the research.

Findings are presented both qualitatively and quantitatively while performing the content analysis in this study. The manner with which the findings are formed into themes, namely the stages of qualitative analysis, is presented above. The following explanations are provided concerning the quantitative dimension of content analysis: although 25 school managers were interviewed in the study, the numbers in the finding tables is greater than 25 because in the scenarios, school managers gave more than one answers to the questions they were asked. In summary, while quantifying the research findings obtained as a result of the content analysis, frequencies and percentages are included; the mentioned frequency and percentage values are not calculated based on the sample, meaning the total number of school managers, but based on the total answers (opinions) given to the questions in each scenario.

## 4. Findings

The findings obtained in this research are presented in the four tables below, with one for each of the scenarios related to the four different human natures.

In the first scenario, the behavior of the analyzed teacher is “A teacher, who has fulfilled the legal weekly hours of class is expected to give 1 extra hour of class because there is a teacher shortage at school. While the teacher refused this at the beginning, he/she accepted this duty when he/she learned that he/she would get overtime pay.” Table 1 shows how teachers with this human nature are defined by administrators as well as their attitudes toward such teachers.

As can be seen in Table 1, teachers with a rational-economic nature were defined by 33.33% of the school administrators as monetarist, or money-grabbing. The administrators supported their opinions with the following statements:

“*He/she is right because he/she fulfilled the weekly class requirement. However, I think he/she is a monetarist because he/she gives importance to money and tries to compensate his/her immaterial loss with monetary gain*.”

(Y-1)

The second most frequently used definitions by administrators were selfish and legally bound, with 25% respectively. Administrators who used the adjective selfish said,

“*I think these teachers are people who do not adhere to the principle of working devotedly and are selfish* because *they know the rules and use them for their own benefit.*”

(Y-12)

“*A selfish person who thinks of his/her own interests more than the students and their education; a person trying to give a rest to him/herself.*”

(Y-18)

As opposed to this, administrators believe that the teachers are legally right with the same percentage, and defined them positively with the following statements:

“*A teacher who is aware that forced labor is a crime under the law, acts in accordance with the law and is* aware *of his/her legal rights.*”

(Y-7)

A total of 13.89% of the administrators defined such teachers as indifferent and non-helpful, while 2.78% of the administrators defined teachers of this nature as people who are acting naturally. Their statements are shown below.

“*He/she does not care about helping the school administration. In addition, they are indifferent to their own* students *losing classes.*”

(Y-3 indifferent person)

“*Considering the personal rights of teachers acting in such a way, their behaviors are quite normal. I believe they have a natural acting personality.*”

(Y-6 natural acting person)

In the second part of Table 1, administrator attitudes toward teachers with a “rational-economic” nature are evaluated. According to this, the administrator attitude with the highest percentage (21.05%) is to respect those teachers, considering that they are trying to protect their legal rights. Again, with the same ratio, administrators expressed that they would show negative reactions to such teachers and try to avoid them. Administrators used the following statements to express their opinions:

“*There won’t be any negativity in my attitude; I’ll display a positive attitude because it is his/her legal right and it should be respected. I show a relatively normal attitude.*”

(Y-14, respectful attitude)

“*I try to stay away from teachers with such an understanding. If necessary, I try to cover up for the loss of* hours *myself, but I don’t let a discussion disrupt the serenity at school.*”

(Y-11, trying to stay away)

These attitudes of administrators are followed by 18.42% for attempting to persuade, 13.16% being indifferent, 7.89% taking on the task, and 7.89% taking sides with the teacher. Administrators supported these attitudes in the following way:

“*I tell him/her that I know he/she has a legal right in this, and that I don’t find it odd. However, since such situations are not frequent, I ask him/her to show understanding on this occasion and to assist the administration.*”

(Y-17, attempt to persuade)

“*I don’t show any reaction because he/she is legally right. I ask if he/she would help, but if the answer is no, I drop the subject and try to find other solutions.*”

(Y-9, being indifferent)

“*The reaction of each* teacher *is different in such situations. If the teacher in such a situation is one who doesn’t work willingly, or is not helpful to the administration, I take on the task myself and drop the subject without further discussion.*”

(Y-11, taking on the task)

“*Not* exceeding *the weekly lesson load is the legal right of the teacher. In that case, I defend the teacher and look for a different solution to the problem.*”

(Y-6, being on the same side with the teacher)

The teacher behavior analyzed in the second scenario, which was given to the administrators to get their opinions, is the following: “A teacher who used to sign the decisions records in previous years stated without any explanation that he/she will no longer sign it”. Table 2 shows how teachers with this human nature are defined by administrators, and what their attitudes are toward these teachers.

Looking at the teachers with “complex” human nature, as shown in Table 2, they are described by the administrators as being irresponsible, with a ratio of 24.24%. This is followed by “inconsistent”, with a ratio of 21.21%. After these two negative descriptions, with an attitude of questioning the reasons for such behaviors, administrators thought those teachers probably had some negative experiences in the past, or were affected by some problems, thus resulting in their complex nature. Administrators defined these three different opinions with high percentages as

“*Such teachers try to escape from the responsibilities given to them; they do not want to take any responsibility.*”

(Y-2, irresponsible)

“*People with this type of behavior are changeable. They act in a manner that suits their interests; they are changeable and inconsistent in their behaviors.*”

(Y-22, inconsistent)

“*The reason for this person’s instability could be some previous negative experience. He/she is a resentful person because of previous negative experiences.*”

(Y-10, having negative experience)

Apart from the above adjectives, administrators described teachers with complex human nature in the following order: 12.12% incompatible, 9.09% low motivation, 6.06% selfish, 3.03% indecisive and 3.03% easily influenced. Examples for these descriptions are given below.

“*In short, I believe these people are incompatible and they resist obeying the rules.*”

(Y-16, incompatible)

“*I describe him/her as someone with little will to work, low motivation, and who does not to want to take responsibility.*”

(Y-4, low motivation)

“*A selfish personality, acting in line with self-benefits.*”

(Y-6, selfish)

“*Hard to explain his/her behaviors and decisions; someone who is indecisive, confused and causes confusion in the workplace.*”

(Y-1, indecisive)

“*Displaying behavior depending on the people around him/her; causing problems by getting under the influence of others.*”

(Y-3, getting under influence)

While the administrator opinions on “complex” human nature are illustrated above, administrator behaviors toward people with such nature are shown in the second section of Table 2. As can be seen in the table, using the law and applying sanctions to teachers with “complex” nature, according to administrator opinions, is in the first place with a high ratio of 41.03%. The second place is shared by attempting to understand the reason for the behavior and trying to help the teacher/solve the problem with an equal ratio of 23.08%. These are followed by attempts to treat all teachers equally, with 10.26%, and finally, remaining unresponsive with 2.56%. Administrators revealed their attitudes in the following statements:

“*It is difficult to make people with such behavior accept anything against the law. Therefore, when I encounter such situations, I act within the law and make sure whatever is necessary gets done.*”

(Y-19, using sanctions)

“*First of all, I call that person who acts like that and try to learn the reason. I tell him/her that we can find a mutual solution if he/she explains the reason.*”

(Y-20, understanding the reason)

“*If he/she is showing that behavior because of a reason, I try to find it out. I call him/her to find out the reason. Later, I try to solve the problem and be helpful.*”

(Y-7, being helpful/attempt to solve the problem)

“*I try to be equal and just toward all. Therefore, if there is a rule or situation that everyone has to follow, I make sure all teachers abide by that rule.*”

(Y-3, effort to be fair)

“*I don’t show any reaction to someone behaving like this. If I show a reaction and apply sanctions, the harmony in the school may be disrupted.*”

(Y-12, unresponsive)

The teacher behavior presented to the administrators in the third scenario was the following:

“Without any expectations, the teacher voluntarily cares about students who are behind in their school work individually, during his/her free time. In addition, he/she develops him/herself by attending educational courses and conferences, without the goal of being promoted.” Table 3 shows how teachers of this human nature are defined by administrators, and the attitudes of administrators toward these teachers.

As shown in Table 3, the most frequently used adjective by the administrators to describe teachers with a “self-realizing” human nature is “unselfish/devoted” by 23.40%, and the following statement supported this:

“*Teachers with such a personality are unselfish people who carry out their work devotedly.*”

(Y-7)

After the adjective “unselfish,” respecting/loving the profession was used by 21.28%. One of the statements given by administrators regarding this adjective is

“*A person who loves his/her profession and children. Doing his/her job with love, not by obligation. There are such teachers in my school too. Both student and parent opinions regarding those teachers are positive to the degree of happiness.*”

(Y-10)

Besides these two most frequently used adjectives, those used by the administrators to describe teachers are ranked in the following order: Inquisitive/interested with 19.15%, model teacher with 12.77%, open to learning with 10.6%, hardworking with 10.64%, and rare person 2.13%. Some opinions of the administrators related to these descriptions are:

“*This is praiseworthy behavior. These are behaviors of teachers who are inquisitive and interested in their students.*”

(Y-20, Inquisitive/interested)

“*A teacher with this personality should be taken as a model by teachers of all ages.*”

(Y-16, Model teachers)

“*A person with an understanding of learning while teaching; developing him/herself together with the students. Open to learning.*”

(Y-23, Open to learning/developing)

“*In one word: Hardworking.*”

(Y-6, Hardworking)

“*What I expect from every teacher, as an administrator and a parent. Unfortunately, teachers with such a personality are rare in our schools.*”

(Y-19, Rare person)

The second part of Table 3 displays the administrator approaches toward “self-realizing” people. At the top of participating administrator approaches toward these teachers was the “I helping/supporting approach”, which was expressed by more than half of the administrators with a ratio of 58.33%. Following this view in order are appreciating/rewarding with 16.67%, being/showing satisfaction with 10.42%, respecting/valuing with 8.33%, and motivating with 6.25%.

“*It’s a very good feeling to work with teachers that exhibit such behavior. There are teachers like this in my school now. I try to help and support them as an administrator, as much as I can.*”

(Y-1, Helping/supporting)

“*I have an extreme feeling of love for such teachers. I dignify and appreciate them everywhere, and I say this at every occasion.*”

(Y-12, Valuing)

“*I feel grateful to such teachers. I always try to be helpful, and always say that I am happy for their work.*”

(Y-5, Being happy)

“*I have respect for teachers like this who love their profession. I always approach them with respect and value their wishes.*”

(Y-22, Respecting)

“*I do anything I can for such teachers. I try to motivate them so they continue working without losing their enthusiasm.*”

(Y-4, Motivating)

In the final scenario given to the administrators, the teacher behavior analyzed is as follows: “After carrying out his/her legal obligations at school, he/she manages the MNE Educational Web page, which is related to administrative work, and helps his/her administrators and colleagues”. Table 4 shows how teachers of this human nature are described by administrators and what their attitudes are toward these teachers.

As can be seen in Table 4, teachers with a “social” nature are defined by administrators as big-hearted/helpful with a ratio of 28.85%. One of the administrators stated this as:

“*A person tries to help the administration, and not only has the responsibility of students in his/her class but is a helpful teacher suitable to take responsibility for other school work.*”

(Y-19)

The second place in administrator opinions is “devoted/self-sacrificing,” and “successful” with 19.23%. Examples of these adjectives are:

“*These teachers believe in ‘we’, not ‘I’. They are people who love doing well and helping others, and they approach their profession with devotion.*”

(Y-23, Self-sacrificing)

“*A person well informed in his/her profession, and successful.*”

(Y-8, Successful)

After these definitions by administrators, interested (13.46%), innovative/enterprising (7.69%), responsible (5.77%), and unselfish/thoughtful (1.92%) appear in order. The following quotes can be given as examples to these definitions:

“*A person who cares for his/her profession and work place, is interested in his/her profession in every aspect, not just in the classroom environment.*”

(Y-11, Interested)

“*A teacher profile open to innovations, helpful, renews his/her knowledge and applies his/her new learnings in his/her profession.*”

(Y-17, Innovative)

“*A person who gives importance to his/her profession, knows how to take responsibility, and carries out his/her responsibilities in the best way possible.*”

(Y-6, Responsible)

“*An unselfish person not withholding his/her time and effort, thinking of his/her institution, not him/herself.*”

(Y-3, Unselfish)

“*In one word: Thoughtful.*”

(Y-1, Thoughtful)

“*A teacher of good personality that every school should have; ready to help the administration.*”

(Y-20, Much needed person)

While the administrators defined teachers with “social” human nature as shown above, they stated their attitudes toward these teachers in the second section of Table 4. According to this, the attitudes of administrators toward such teachers are, in descending order: Helping/supporting (47.06%), Appreciating/rewarding (27.45%), Showing satisfaction (3.92%), and Respecting (1.96%).

“*I give every chance necessary to a teacher like this. I try to make things easier for them.*”

(Y-7, Helping/supporting)

“*I appreciate such a teacher for taking part in the administrative aspects of the school voluntarily; I try to reward him/her appropriately to ensure that he/she continues his/her work.*”

(Y-16, Appreciating/rewarding)

“*I thank him/her for his/her contributions. I support him/her in his/her classwork when he/she is in a tight position or does not feel well.*”

(Y-19, Showing satisfaction)

“*I give support to such a teacher in the things he/she wants to do. I praise and honor him/her. I try to make sure that both I and other teachers benefit from his/her knowledge.*”

(Y-10, Gaining advantage/benefiting)

“*I have great love and respect for a teacher of this personality. I support him/her in whatever he/she wants to do and show my satisfaction and respect whenever possible.*”

(Y-25, Respecting)

## 5. Discussion and Conclusions

In this study, it is believed that awareness will be raised concerning the necessity of school management focused on teachers. In such structures as Northern Cyprus, which are dominated by a centralized education system, the education policies of the Ministry of National Education and the government are usually given priority instead of the necessities of teachers and students. However, administration should be a people-oriented activity and according to Hofstede, administrators cannot manage people unless they understand their values, beliefs, the terms they use, and empathize with them [41,42,43,44]. Every person shows differences within him/herself, and it is vital to manage these differences in order to sustain administrative activities. According to Thomas [44], managing differences is not about controlling them, but valuing the differences and similarities of employees, and discovering their potential and the contributions they can provide toward the goals of the institution. From this perspective, it is of significant importance that a good school administrator knows the teachers in the organization and designates his/her attitude toward them after identifying them. This importance is accompanied by the notion of administrators knowing and defining human resources in their organizations.

The thorough understanding of the human resources in an organization will facilitate the process of distinguishing individuals with their different qualities and predicting how they will behave under different circumstances [45]. Knowing a person means knowing his/her psychological, biological, and sociological behaviors, personal characteristics, and needs [46]. When discussing the importance of knowing a person and having information about the nature of that person, Dilthey mentioned the presence of the nature that each person has and claimed that each person is an organization within him/herself [47]. It is widely acknowledged getting to know a person is a difficult process. Researchers have identified that in order to know a person, it is essential that an administrator knows themselves first [43]. Afterward, it is necessary to gather data about the person and use skills such as analysis and interpretation [48].

As stated in this study in general, before being able to perform the important activities for the organization, such achieving organization goals and ensuring success within the organization, it is imperative that the administrator knows him/herself and the employees. Hence, if school administrators want the school to be successful and reach its goals, they have to get to know the staff under their management. Based on this fact, the following conclusions were reached on the topic of administrators getting to know human nature and designating suitable attitudes.

The human natures of teachers seen as positive characteristics by the school administrators are self-realizing and social human nature types. Administrators described teachers of these natures with positive adjectives in general (self-sacrificing-unselfish, hardworking, model teacher, bighearted, successful, thoughtful etc.). However, when describing teachers with a rational-economic human nature, it was observed that there was no consensus among the administrators. For example, while some administrators used adjectives such as “selfish, money grabbing, not helpful” for teachers with economic human nature, other administrators described them as “a person abiding by the law or acting natural”. Looking at the four human natures within the scope of this research, it was found that the complex human nature attracted the most negativity. Teachers of complex human nature were described by their administrators as “irresponsible, incompatible, with low motivation”. The reason for this could be that inconsistent, changeable, indecisive people (that is, teachers with complex human nature) are very difficult to manage. On the other hand, when teachers were asked about their perceptions of their own human natures, they stated that they did not perceive themselves as having complex human nature [17,49], and this finding is noteworthy. Again, looking at the adjectives used by administrators, it was observed that the most positive adjectives were used for people with self-realizing human nature. Another conclusion of this research which should be mentioned is that teachers with a self-realizing nature are defined in a more positive way compared to those with social human nature, to the extent that school administrators used adjectives such as “rare (valuable), model, self-sacrificing, respectful of profession, and hardworking” for teachers with self-realizing human nature. In the research findings of Kalafat, while describing their personal characteristics, teachers assessed themselves as extroverts, open to gain new experiences, and aware of their responsibilities [50]. Conclusions can be found in the literature that professional competence is directly related with positive personal characteristics (having responsibility, being emotionally stable) [51,52]. The most remarkable conclusion of this study is the fact that the efforts of school managers to influence the personality characteristics of teachers with whom they work in a positive direction are limited. The school managers who were interviewed in the study did not define how they will earn and develop some of the teachers they described as “problematic”. Therefore, it became clear that the mentioned school principals respected school-based management and the professional or personal development of teachers was not one of their objectives.

Other findings of the research are related to the management of human resources. Human resources management is a notion which enables an organization to reach its goals [53], affects the sustainability of organizations [54], and contributes to the effective use of physical and financial sources of organizations [55]. In this research, human resources management is assessed by the attitudes of administrators toward the behaviors that individuals display, and toward the human natures they have as well as their attitudes. Hence, looking at the findings of the research, the attitudes of school administrators toward teachers with different human natures can be grouped under different headings. The attitudes of school administrators were primarily toward using leadership skills and aiming to apply effective management; in other words, standing by the teachers. On the other hand, the presence of negative administrator behaviors was observed, such as punishing, being indifferent and only applying the laws. Attitudes of punishment were observed to be behaviors like giving negative reactions, avoiding and applying sanctions. School administrators display effective behaviors (helping, appreciating and rewarding, showing satisfaction, respecting, benefiting, and motivating) and positive leadership characteristics toward teachers with self-realizing and social human natures. School administrators stated that they always support teachers with this human nature. There are differences in terms of the attitudes of school administrators toward teachers with economic and complex human natures. As mentioned above, administrators are either indifferent or they apply sanctions. On the other hand, there are school administrators who limit themselves to merely applying the laws. School manager behaviors which vary depending on teacher behaviors can lead to the emergence of some problems in school management. For example, teachers may think that discrimination is being made against their colleagues and fail to make sense of the behaviors of school managers. Thus, instead of the school management creating a competitive environment, it may be more beneficial to produce solutions without individualizing problems and ensuring group awareness and sensitiveness. At the beginning of school period in particular, it is essential that the tasks expected from teachers are clearly defined and no concessions are made in this regard by school principals.

The most important limitation of this study is that only the opinions of school managers were consulted while determining the opinions concerning human nature. In fact, if the opinions of teachers could also be received and evaluated, a two-fold contribution could be made to the literature. First, teachers could be allowed to make self-criticisms concerning their own human nature. Second, the opinions of school managers could be compared with teacher opinions and discussed. On the other hand, the most important contribution of this study is that the scenarios written by researchers provided a favorable ground for school managers to make comments and express their opinions easily. Instead of collecting data through interviews and observations and describing real situations, fictional scenarios have been used in this study. Resultantly, based on fictional scenarios, opinions have been obtained from school managers in a creative, free and natural process. School managers expressed their opinions without being concerned about who might hear what they said or who could have been insulted. Indeed, the fact that several citations are made from the opinions of school managers in the “findings” section and that these citations are given in detail is an indicator of this situation.

## 6. Recommendations

Looking at the research findings, it can be said that there are school administrators who undertake the duties themselves. Based on the research findings, the following recommendations are presented:

1. Considering that school administrators understand the human nature characteristics of teachers, they can define the personal characteristics of teachers in detail, and they should use their findings in school administration. Thus, a more productive process of administration will be realized in the operation of the school and in teacher assignments.
2. It was found that teachers with self-realizing human nature are positively assessed by school administrators. Therefore, it can be recommended that school administrators provide suitable conditions and environments for these teachers to improve themselves and contribute to the school.
3. Teachers with complex human nature are perceived by school administrators as teachers causing problems. In this case, school administrators need to improve their skills in managing differences through in-service training.
4. Teachers with social human nature are defined by school administrators as helpful. Teachers with this nature are people who can be supportive to administrators in improving communication at school. They can be useful in creating bonds between teachers and the school administrator, thus improving interactions at the school.

It was observed in the research that administrators have a tendency to avoid or be unresponsive to teachers with rational-economic human nature and give jobs to others. It is recommended that the causes behind such attitudes should be examined, and new research should be conducted related to this topic.

## Figures and Tables

**Table 1:** “Rational-economic” teachers in the eyes of school administrators.

	Teachers with “Rational-Economic” Human Nature		
Themes	Sub-Themes	*N*	%
Defined by administrators as	Giving importance to money-monetarist-money grabbing	12	33.33
	Selfish	9	25
	Sticking to rules	9	25
	Non helpful-indifferent	5	13.89
	Acting naturally	1	2.78
	**TOTAL**	**36**	**100**
Attitude toward teachers	Thinking it’s their legal right—respecting	8	21.05
	Showing negative response/attitude—trying to stay away	8	21.05
	Attempt to persuade—using power of leadership	7	18.42
	Being indifferent	5	13.16
	Applying sanctions	4	10.53
	Taking the task on—giving the task to others	3	7.89
	Taking sides with the teacher	3	7.89
	**TOTAL**	**38**	**100**

**Table 2:** “Complex” teachers in the eyes of school administrators.

	Teachers with “Complex” Human Nature		
Themes	Sub-Themes	*N*	%
Defined by administrators as	Irresponsible	8	24.24
	Changeable-inconsistent	7	21.21
	Having negative experience-problematic	7	21.21
	Incompatible	4	12.12
	With low motivation	3	9.09
	Selfish	2	6.06
	Indecisive	1	3.03
	Easily influenced	1	3.03
	**TOTAL**	**33**	**100**
Attitude of administrators toward them	Sanctions-using the law	16	41.03
	Trying to understand the reason	9	23.08
	Trying to help-persuading-attempting to solve the problem	9	23.08
	Trying to be equal toward all teachers	4	10.26
	Being unresponsive	1	2.56
	**TOTAL**	**39**	**100**

**Table 3:** “Self-realizing” teachers in the eyes of school administrators.

	Teachers with “Self Realizing” Human Nature		
Themes	Sub-Themes	*N*	%
Defining the teacher	Unselfish-devoted	11	23.40
	Respecting and loving the profession	10	21.28
	Inquisitive-interested	9	19.15
	Model teacher	6	12.77
	Open to learning/development	5	10.64
	Hardworking	5	10.64
	Rare	1	2.13
	**TOTAL**	**47**	**100**
Attitude toward the teacher	Helpful-supporting	28	58.33
	Appreciating-rewarding	8	16.67
	Being satisfied-showing satisfaction	5	10.42
	Respecting-valuing	4	8.33
	Motivating	3	6.25
	**TOTAL**	**48**	**100**

**Table 4:** “Social” teachers in the eyes of school administrators.

	Teachers with “Social” Human Nature		
Themes	Sub-Themes	*N*	%
Defined by administrators as	Bighearted-helpful	15	28.85
	Devoted-self sacrificing	10	19.23
	Successful	10	19.23
	Interested	7	13.46
	Innovative-enterprising	4	7.69
	Responsible	3	5.77
	Unselfish	1	1.92
	Thoughtful	1	1.92
	Much needed person	1	1.92
	**TOTAL**	**52**	**100**
Attitudes toward these teachers	Helping-supporting	24	47.06
	Appreciating-rewarding	14	27.45
	Showing satisfaction	10	19.61
	Gaining advantage-benefiting	2	3.92
	Respecting	1	1.96
	**TOTAL**	**51**	**100**

## References

[1] Taymaz H. (2009). İlköğretim ve Ortaöğretim Okul Müdürleri Için Okul Yönetimi.

[2] Bursalıoğlu Z. (2010). Eğitim Yönetiminde Yeni Yapı ve Davranış.

[3] Demirtaş H., Güneş H. (2012). Eğitim Yönetimi ve Denetimi Sözlüğü.

[4] Gelsthorpe T., West-Burnham J. (2003). Educational Leadership and the Community: Strategies for School Improvement through Community Engagement.

[5] Şişman M. (2002). Öğretim Liderliği.

[6] Bursalıoğlu Z. (1994). New Structures and Behaviors in School Management.

[7] Turan S. (2015). Eğitim Yönetimi, Teori, Araştırma ve Uygulama.

[8] Mayo E. (1945). The Social Problems of an Industial Civilization.

[9] Şişman M. (1994). Örgüt Kültürü.

[10] Çağlayan E. (2001). İlköğretim Okullarında Görev Yapan Yöneticilerin Insan Doğasına Ilişkin Algıları (Bursa ili Örneği). Master’s Thesis.

[11] Wrightsman L.S., Wuescher M.L. (1974). Assumptions about Human Nature: A Social-Psychological Approach.

[12] Aydın M. (2014). Eğitim Yönetimi.

[13] Durukan H. (2003). Yönetimde insan ilişkileri. Kastamonu Eğitim Dergisi.

[14] İlgar L. (1996). Eğitim Yönetimi, Okul Yönetimi, Sınıf Yönetimi.

[15] Tuncer P. (2012). Değişen insan kaynakları yönetimi anlayışında kariyer yönetimi. Ondokuz Mayıs Üniversitesi Eğitim Fakültesi Dergisi.

[16] Özçelik F. (2002). Yöneticilerin Insan Doğasına Ilişkin Görüşleri ve Yönetsel Yaklaşımlar.

[17] Mete C. (2006). İlköğretim Okullarında Çalışan Öğretmenlerin Kişilik Özellikleri Ile Iş Tatminleri Arasındaki Ilişkinin Incelenmesi. Master’s Thesis.

[18] Russell B., Göktürk A. (1997). Din Ile Bilim.

[19] Küçükkaragöz H. (1992). Sosyal bir ünite olarak okul içi etkileşimi. Hacettepe Üniversitesi Eğitim Fakültesi Dergisi.

[20] Bilgin N. (1988). Sosyal Psikolojiye Giriş.

[21] Kılıçbay A. (1983). Uygulamalı Ekonometri.

[22] MacGregor D. (1960). The Human Side of Enterprise.

[23] Turan S., Şişman M. (2000). Okul yöneticileri için standartlar: Eğitim yöneticilerinin bilgi temelleri üzerine düşünceler. Balıkesir Üniversitesi Sosyal Bilimler Enstitüsü Dergisi.

[24] Schein E., Tosun M. (1978). Örgüt Psikolojisi.

[25] Everard K.B., Morris G., Wilson I. (2004). Effective School Management.

[26] Fromm E., Doğangün A. (1998). İnsan Bilgisi ve Hümanist Planlama.

[27] Ouchi W.G., Güneri Y. (1987). Teori Z: Japonların Yönetim Tarzı Nasıl İşliyor?.

[28] Nephan S. (1989). Antropoloji.

[29] Eren E. (1989). Yönetim Psikolojisi.

[30] Taylor-Gooby P. (1981). Part I: Approaches to Welfare State, Social Theory and Social Welfare.

[31] Efil İ. (1996). İşletmelerde Yönetim ve Organizasyon.

[32] Yıldırım A., Şimşek H. (2013). Sosyal Bilimlerde Nitel Araştırma Yöntemleri.

[33] Ju X.T., Xing G.X., Chen X.P., Zhang S.L., Zhang L.J., Liu X.J., Cui Z.L., Yin B., Christie P., Zhu Z.L. (2009). Reducing environmental risk by improving N management in intensive Chinese agricultural systems. Proc. Natl. Acad. Sci. USA.

[34] Emiroğlu O., Güneyli A., Burgul N.S. (2017). Motivational sources of teachers in a developing country. Revista de Cercetare si interventie sociala..

[35] OECD Higher Education to 2030: Volume I–II, 2009. https://www.oecd-ilibrary.org/education/higher-education-to-2030-volume-2-globalisation_9789264075375-en.

[36] Saussois J.M. (2006). Scenarios, international comparisons, and key variables for educational scenario analysis. Think Scenarios, Rethink Education.

[37] Schoemaker P.J. (1995). Scenario planning: A tool for strategic thinking. Sloan Manag. Rev..

[38] Williams J.A., McNeil K.R. (2010). Using Scenario Analysis to Build University Faculty and Student Travel Competencies. http://www.aabri.com/manuscripts/09395.pdf.

[39] Balcı A. (2004). Türkiye’de eğitim yöneticisi ve eğitim müfettişi yetiştirme uygulamaları: Sorunlar ve öneriler. Çağdaş Eğitim Dergisi.

[40] Miles H.B., Huberman A.M. (1994). Qualitative Data Analysis: An Expanded Sourcebook.

[41] Tikici M., Türk M. (2003). İnsan odaklı yönetim ve müşteri memnuniyeti: Malatya ilinde bir uygulama. Süleyman Demirel Üniversitesi İktisadi ve idari Bilimler Fakültesi Dergisi.

[42] Hofstede G. (1984). Cultural dimensions in management and planning. Asia Pac. J. Manag..

[43] Baltaş A. (2013). Türk Kültüründe Yönetmek.

[44] Sürgevil O., Budak G. (2008). İşletmelerin farklılıkların yönetimi anlayışına yaklaşım tarzlarının saptanmasına yönelik bir araştırma. Dokuz Eylül Üniversitesi Sosyal Bilimler Enstitüsü Dergisi.

[45] Aktepe V. (2005). Eğitimde bireyi tanımanın önemi. Gazi Üniversitesi Kırşehir Eğitim Fakültesi Dergisi.

[46] Yeşilyaprak B., Güngör A., Kurç G. (1996). Eğitsel ve Mesleki Rehberlik.

[47] Dilthey W., Özlem D. (1999). Hermeneutik ve Tin Bilimleri.

[48] Özgüven İ.E. (1998). Bireyi Tanima Teknikleri.

[49] Ekşi H. (2004). Kişilik ve başaçıkma: Başaçıkma tarzlarının durumsal ve eğilimsel boyutları üzerine çok yönlü bir araştırma. Kuram ve Uygulamada Eğitim Bilimleri.

[50] Kalafat S. (2012). Ortaöğretim Öğretmenlerinin Kişilik Özelliklerinin Öğretmen Yeterliliklerine Etkisinin Çeşitli Değişkenler Açısından Incelenmesi. Master’s Thesis.

[51] Burger J.M., Deniz İ., Sarıoğlu E. (2006). Kişilik: Psikoloji Biliminin Insan Doğasına Dair Söyledikleri.

[52] Hartman R.O., Betz N.E. (2007). The five-factor model and career self-efficacy general and domain-specific relationships. J. Career Assess..

[53] Aykaç B. (1999). İnsan Kaynakları Yönetimi ve Insan Kaynaklarının Stratejik Planlaması.

[54] Çalık T. (2003). Öğrenen örgütler olarak eğitim kurumları. Manas Üniversitesi Sosyal Bilimler Dergisi.

[55] Palmer M., Winters K.T. (1993). İnsan Kaynakları.

